# The Core Splicing Factors EFTUD2, SNRPB and TXNL4A Are Essential for Neural Crest and Craniofacial Development

**DOI:** 10.3390/jdb10030029

**Published:** 2022-07-08

**Authors:** Byung-Yong Park, Melanie Tachi-Duprat, Chibuike Ihewulezi, Arun Devotta, Jean-Pierre Saint-Jeannet

**Affiliations:** Department of Molecular Pathobiology, College of Dentistry, New York University, 345 East 24th Street, New York, NY 10010, USA; parkb@jbnu.ac.kr (B.-Y.P.); mel.td31@gmail.com (M.T.-D.); ci13@nyu.edu (C.I.); ad146@nyu.edu (A.D.)

**Keywords:** *Xenopus*, neural crest, craniofacial, mandibulofacial dysostosis, apoptosis, spliceosome, Sf3b4, Eftud2, Snrpb, Txnl4a

## Abstract

Mandibulofacial dysostosis (MFD) is a human congenital disorder characterized by hypoplastic neural-crest-derived craniofacial bones often associated with outer and middle ear defects. There is growing evidence that mutations in components of the spliceosome are a major cause for MFD. Genetic variants affecting the function of several core splicing factors, namely *SF3B4*, *SF3B2*, *EFTUD2*, *SNRPB* and *TXNL4A*, are responsible for MFD in five related but distinct syndromes known as Nager and Rodriguez syndromes (NRS), craniofacial microsomia (CFM), mandibulofacial dysostosis with microcephaly (MFDM), cerebro-costo-mandibular syndrome (CCMS) and Burn–McKeown syndrome (BMKS), respectively. Animal models of NRS and MFDM indicate that MFD results from an early depletion of neural crest progenitors through a mechanism that involves apoptosis. Here we characterize the knockdown phenotype of Eftud2, Snrpb and Txnl4a in *Xenopus* embryos at different stages of neural crest and craniofacial development. Our results point to defects in cranial neural crest cell formation as the likely culprit for MFD associated with *EFTUD2*, *SNRPB* and *TXNL4A* haploinsufficiency, and suggest a commonality in the etiology of these craniofacial spliceosomopathies.

## 1. Introduction

Mandibulofacial dysostosis (MFD), also known as Treacher Collins syndrome (TCS; OMIM#154500), is a rare human congenital disorder characterized by underdeveloped craniofacial bones. The main features of the disease include hypoplasia of the zygomatic complex, micrognathia, downslanting palpebral fissures, coloboma of the lower eyelid, and outer and middle ear defects [1]. The condition arises from abnormal development of the first and second pharyngeal arches and their derivatives [2,3]. All forms of MFD present with the same craniofacial anomalies and may have additional features, such as limb defects, microcephaly, intellectual disability or heart defects, which distinguish them clinically [4].

The spliceosome is a complex of proteins and RNAs involved in the processing of pre-mRNAs into mature RNAs through the removal of introns [5]. Mutations in genes encoding proteins of the spliceosome cause diseases referred to as spliceosomopathies. In recent years, mutations in genes encoding core components of the major spliceosome have been linked to several forms of MFD [6,7,8,9]. These genes include *SF3B4*, *SF3B2*, *EFTUD2*, *SNRPB*, *TXNL4*, *PUF60*, *EIF4A3* and *CWC27*. Genetic variants for these factors cause Nager and Rodriguez syndromes (NRS; OMIM#154400 and OMIM#201170), craniofacial microsomia (CFM; OMIM#605591), mandibulofacial dysostosis with microcephaly (MFDM; OMIM#610536), cerebro-costo-mandibular syndrome (CCMS; 608572), Burn–McKeown syndrome (BMKS; OMIM#608572), Verheij syndrome (VS; OMIM#615583), Richieri–Costa–Pereira syndrome (RCPS; OMIM#268305) and retinitis pigmentosa with or without skeletal anomalies (RPSKA; OMIM#250410), respectively.

It is intriguing that disruption of the spliceosome machinery, which is expected to be active in all cell types, results in defects affecting only a limited number of lineages in the adult, as seen in retinitis pigmentosa, myelodysplastic syndromes and craniofacial disorders. The cellular and molecular bases for the tissue-/cell-type-specific pathologies caused by these mutations remain largely unknown [8]. Animal models of TCS [10], NRS [11] and MFDM [12,13] have started to investigate the mechanisms underlying the etiology of MFD. These studies suggest that MFD results from an early depletion of neural crest progenitors, the cell type that populates the pharyngeal arches and forms most structures of the face, through a mechanism that involves increased cell death, resulting in craniofacial abnormalities.

To determine whether MFDs share a common root cause across multiple craniofacial spliceosomopathies we have analyzed the function of *Eftud2*, *Snrpb* and *Txnl4a* in *Xenopus* embryos using a morpholino-based knockdown approach. Our results show that interference with these factors causes altered gene expression in pre-migrating and migrating neural crest cells that correlates with increased apoptosis in the ectoderm. Later in development, these animals exhibited defects in neural-crest-derived craniofacial cartilages. We propose that MFD associated with *EFTUD2*, *SNRPB* and *TXNL4A* haploinsufficiency has a common root cause, suggesting a universal mechanism underlying the etiology of craniofacial spliceosomopathies.

## 2. Materials and Methods

### 2.1. Xenopus Embryos, Constructs and Microinjections

*Xenopus laevis* embryos were staged according to Nieuwkoop and Faber [14] and raised in 0.1X NAM (Normal Amphibian Medium; [15]). The work was approved by the Institutional Animal Care and Use Committee of New York University (protocol #IA16-00052). Splicing factor constructs, pSPORT6-*eftud2.S*, pExpress1-*snrpb.L* and pBSK-*txnl4a*.S, were purchased from Horizon Discovery (PerkinElmer, Waltham, MA, USA). Standard control (CoMO) and *Eftud2.S* (Eftud2MO: ACTCATCGTACAAGTCAGTGTCCAT), *Snrpb.L* (SnrpbMO: TTGCTGCTTTTTCCCACCGTCATG) and *Txnl4a.S* (Txnl4MO: GATGTGGAAGCATGTACGACATTTC) morpholino antisense oligonucleotides (MOs) were purchased from GeneTools (Philomath, OR). All MOs were injected in one blastomere at the two-cell stage and embryos were analyzed by in situ hybridization (ISH) at the neurula (NF stage 15) or tailbud (NF stage 25) stages. Embryos were co-injected with 500 pg of *LacZ* mRNA to identify the injected side. The optimal dose of MO injected in the embryos at the two-cell stage was determined as the dose for which a phenotype was observed without compromising survival.

For rescue experiments, we used three constructs resistant to the MOs. Human SNRPB (HuSNRPB) and human EFTUD2 (HuEFTUD2) cloned into pCS2+ were purchased from GenScript (Piscataway, NJ, USA). For Txnl4a, we generated by PCR a construct encoding *Xenopus txnl4a* with a modified sequence, inserting 9 mismatched base pairs (underlined), without altering the amino acid composition, using forward and reverse primers (forward: ATCGATGCCACCATGAGTTATATGTTACCGCACCTG, and reverse: TCTAGATCAGTATCTGTACTTGGTGGAATA), and pBSK-*txnl4a*.S as template. The PCR product was cloned into the ClaI and XbaI sites of pCS2+ and sequenced.

### 2.2. Whole-Mount In Situ Hybridization

Embryos were fixed in MEMFA (0.1 M MOPS, 2 mM EGTA, 1 mM MgSO_4_ and 3.7% formaldehyde), stained for Red-Gal (Research Organics; Cleveland, OH, USA) to visualize the lineage tracer (*LacZ*) and processed for ISH. Antisense digoxygenin-labeled probes (Genius kit; Roche, Indianapolis, IN, USA) were synthesized using template cDNA encoding eftud2 (pSPORT6-*eftud2*), snrpb (pExpress1-*snrpb*), txnl4a (pBSK-*txnl4a*), snai2 [16], tfap2e [17], sox9 [18], sox10 [19], sox2 [20] and six1 [21]. Whole-mount ISH was performed as described [22,23].

### 2.3. RT-PCR Analysis

Total RNAs were extracted from embryos using the RNeasy Micro Kit (Qiagen; Valencia, CA, USA). The RNA samples were digested with RNase-free DNase I before RT-PCR. The amount of RNA isolated was quantified by measuring the optical density using a Nanodrop spectrophotometer (Nanodrop Technologies; Wilmington, DE, USA). A One-Step RT-PCR kit (Qiagen; Valencia, CA, USA) was used on a C1000 ThermoCycler (BioRad; Hercules, CA) using primers for *odc* and *sox10* [24], *eftud2* (F:CCCGTCTAATGGAACCCTATTATT; R: CTGAGTTACATGCCCTCTTCTT), *snrpb* (F:GGACCATCACAGCAGGTTAT; R:TGCCCATTGGTGGAAGTAG) and *txnl4a* (F:TGACCCTTGTACAGTGATGTTT; R:CTTGTCTTCCATTGTCCAGTTTATC). The PCR conditions were as follows: denaturation 95 °C (30 s), annealing 55 °C (30 s), extension at 60 °C (30 s) for 30 cycles.

### 2.4. Alcian Blue Staining

Stage 45 tadpoles were fixed in MEMFA for 1 h, rinsed in tap water, dissected, skinned and eviscerated. Tadpole heads were then dehydrated and stained in 0.06% Alcian blue (Sigma-Aldrich; St. Louis, MO, USA) for 12 h. After several washes in 95% ethanol they were rehydrated in 2% potassium hydroxide. Specimens were then transferred in increasing concentrations of glycerol (20%, 40%, 60% and 80%) in 2% potassium hydroxide. The ethmoid plate was dissected and photographed on a Nikon SZX9 stereomicroscope.

### 2.5. TUNEL Assay

TUNEL staining was performed as described [25]. Briefly, MO-injected albino embryos at stage 15 were fixed in MEMFA, rehydrated and washed in TdT buffer (Roche, Indianapolis, IN, USA) for 30 min. End labeling was carried out overnight at room temperature in TdT buffer containing 0.5 μM DIG-dUTP and 150 U/mL TdT (Roche, Indianapolis, IN, USA). Embryos were then washed for 2 h at 65 °C, incubated with Fab anti-DIG antibody conjugated to alkaline phosphatase (Roche, Indianapolis, IN, USA; 1:2000) overnight at 4 °C, and the chromogenic reaction performed using NBT/BCIP (Roche, Indianapolis, IN, USA). To evaluate changes in cell death, embryos were individually photographed and the number of TUNEL-positive cells in the dorsal ectoderm was counted manually, comparing control and injected sides for each embryo.

### 2.6. TNT Reaction

The reaction was performed using the TNT^R^ Quick Coupled Transcription/Translation Systems (Promega, Madison, WI, USA) according to the manufacturer’s instructions using 1 μg of expression construct without or with increasing doses of the corresponding MO (10 ng, 100 ng and 500 ng). The reaction was resolved on a 10% NuPAGE Bis-Tris gel, transferred onto a PVDF membrane using the iBlot system (Invitrogen) and the products revealed using a colorimetric reaction.

### 2.7. Statistical Method

Each experiment was performed on at least two different batches of embryos obtained from different females. In MO-injected embryos we compared gene expression on the injected side with the contralateral uninjected side, and with CoMO-injected embryos. Significance testing for gene expression by ISH was performed using the Chi-squared test, with a *p*-value < 0.05 considered significant. For TUNEL staining, Student’s *t*-test was used to define statistically significant differences between control and injected sides for each MO.

## 3. Results

### 3.1. Developmental Expression of Eftud2, Snrpb and Txnl4a

**Figure 1:**
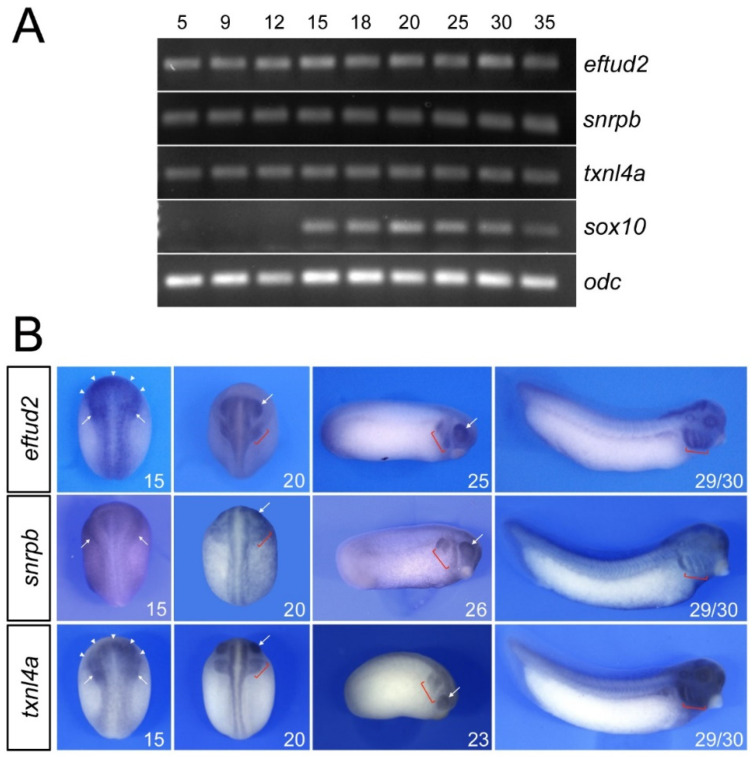
Developmental expression of *eftud2*, *snrpb* and *txnl4a*. (**A**) RT-PCR analysis indicates that *eftud2*, *snrpb* and *txnl4a* are expressed at all stages examined whereas *sox10* is first activated at stage 15. *odc* is shown as a loading control. (**B**) At stage 15, *eftud2* and *txnl4a* transcripts are enriched at the anterior neural plate (arrowheads) and the neural-crest-forming regions (arrows), whereas *snrpb* appears to be ubiquitously expressed. At stages 20 to 26, the three genes are enriched in the pharyngeal arches (brackets) and the eyes (arrows). At stage 29/30, the expression of *eftud2*, *snrpb* and *txnl4a* is maintained in the pharyngeal arches (brackets). Stages 15 and 20 are dorsal views, anterior to top. Stages 23, 25, 26 and 29/30 are lateral views, dorsal to top, anterior to right.

RT-PCR analysis of the developmental expression of *eftud2*, *snrpb* and *txnl4a* indicates that these factors are maternally expressed (stage 5) and are maintained at relatively constant levels throughout development (Figure 1A). By comparison, the neural-crest-specific gene *sox10* is activated at the neurula stage (stage 15; Figure 1A). By whole-mount in situ hybridization, at the neurula stage (stage 15), *eftud2* and *txnl4a* are enriched at the anterior neural plate and the neural-crest-forming regions, in a pattern reminiscent to that of *sf3b4* [11], whereas snrpb appears to be ubiquitously expressed (Figure 1B). By the end of neurulation (stage 20), all three genes show expression in the prospective eyes, migrating cranial neural crest cells and the central nervous system (Figure 1B). At the tailbud stage (stage 23/25), their expression persists in the developing eyes and neural crest streams, and at stage 29/30, *eftud2*, *snrpb* and *txnl4a* transcripts are most abundant in the pharyngeal arches and the head (Figure 1B).

### 3.2. Eftud2, Snrpb and Txnl4a Knockdown Affect Neural Crest Formation

**Figure 2:**
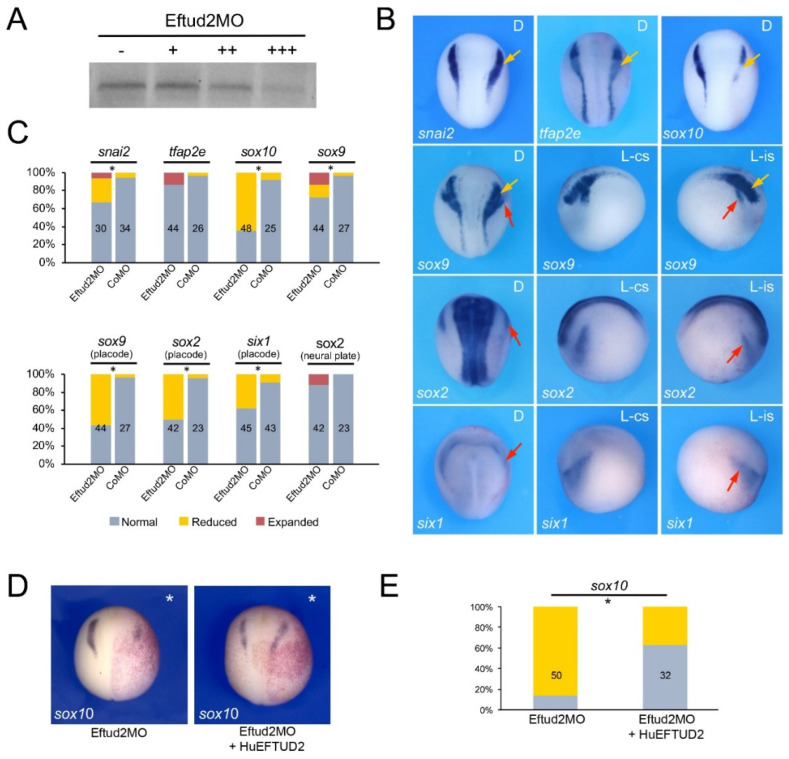
Eftud2 knockdown affects neural crest formation. (**A**) Increasing amounts of Eftud2MO, 10 ng (+), 100 ng (++) and 500 ng (+++), block translation directed by *eftud2* mRNA. (-) Translation without Eftud2MO (**B**) Phenotype of Eftud2MO-injected embryos (30 ng) on neural crest (yellow arrows) and placode (red arrows) gene expression at stage 15. The gene analyzed in each panel is indicated in the lower left corner. D indicates dorsal view, anterior to top. L-cs indicates lateral view control side, anterior to left. L-is indicates lateral view injected side, anterior to right. (**C**) Quantification of the phenotypes. The numbers in each bar indicate the number of embryos analyzed. (**D**) *sox10* expression in Eftud2MO-injected embryos is partially restored by expression of HuEFTUD2 resistant to the MO. The injected side is indicated by an asterisk. Dorsal views, anterior to top. (**E**) Quantification of the results. The numbers in each bar indicate the number of embryos analyzed. Statistically significant differences are indicated (*) *p*-value < 0.05 (Chi-squared test).

**Figure 3:**
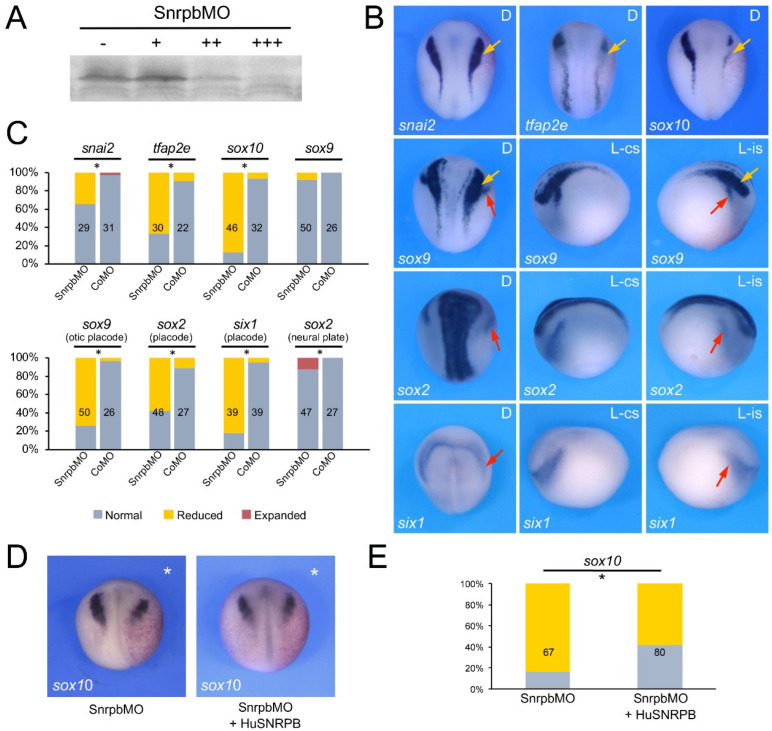
Snrpb knockdown affects neural crest formation. (**A**) Increasing amounts of SnrpbMO, 10 ng (+), 100 ng (++) and 500 ng (+++), block translation directed by *snrpb* mRNA. (-) Translation without SnrpbMO (**B**) Phenotype of SnrpbMO-injected embryos (5 ng) on neural crest (yellow arrows) and placode (red arrows) gene expression at stage 15. The gene analyzed in each panel is indicated in the lower left corner. D indicates dorsal view, anterior to top. L-cs indicates lateral view control side, anterior to left. L-is indicates lateral view injected side, anterior to right. (**C**) Quantification of the phenotypes. The numbers in each bar indicate the number of embryos analyzed. (**D**) *sox10* expression in SnrpbMO-injected embryos is partially restored by expression of HuSNRPB resistant to the MO. The injected side is indicated by an asterisk. Dorsal views, anterior to top. (**E**) Quantification of the results. The numbers in each bar indicate the number of embryos analyzed. Statistically significant differences are indicated (*) *p*-value < 0.05 (Chi-squared test).

**Figure 4:**
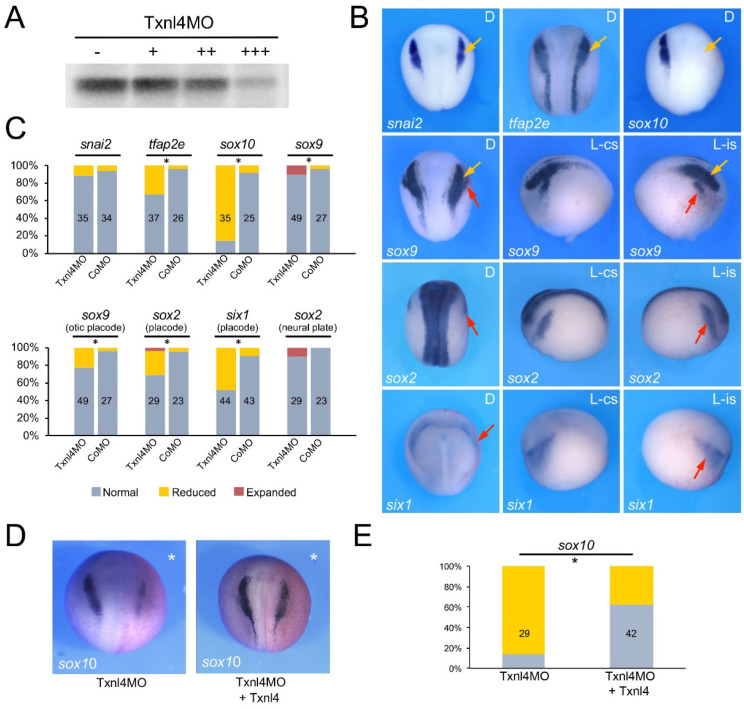
Txnl4a knockdown affects neural crest formation. (**A**) Increasing amounts of Txnl4MO, 10 ng (+), 100 ng (++) and 500 ng (+++), block translation directed by *txnl4a* mRNA. (-) Translation without Txnl4MO. (**B**) Phenotype of Txnl4MO-injected embryos (30 ng) on neural crest (yellow arrows) and placode (red arrows) gene expression at stage 15. The gene analyzed in each panel is indicated in the lower left corner. D indicates dorsal view, anterior to top. L-cs indicates lateral view control side, anterior to left. L-is indicates lateral view injected side, anterior to right. (**C**) Quantification of the phenotypes. The numbers in each bar indicate the number of embryos analyzed. (**D**) *sox10* expression in Txnl4MO-injected embryos is partially restored by expression of *Xenopus* Txnl4 resistant to the MO. The injected side is indicated by an asterisk. Dorsal views, anterior to top. (**E**) Quantification of the results. The numbers in each bar indicate the number of embryos analyzed. Statistically significant differences are indicated (*) *p*-value < 0.05 (Chi-squared test).

To evaluate the function of Eftud2, Snrpb and Txnl4a in the context of neural crest and craniofacial development we performed knockdown of each gene using morpholino antisense oligonucleotides (MO) and analyzed the consequence on neural crest gene expression at the neurula stage (NF stage 15). The MOs were designed to broadly interfere with translation of the corresponding mRNA, and as such are not directly modeling the variant proteins linked to these spliceosomopathies. The efficacy of each translation blocking MOs was tested in an in vitro transcription/translation assay directed by the corresponding expression construct and increasing doses of MOs (Figure 2A, Figure 3A and Figure 4A). Upon unilateral injection of each MO, we found that at the neurula stage *sox10* was the neural crest gene the most severely downregulated in all three morphant embryos, although with different frequency: 64.6% for Eftud2 (Figure 2B,C), 87% for Snrpb (Figure 3B,C) and 85.7% for Txnl4a (Figure 4B,C) knockdowns. Snrpb knockdown also resulted in a marked reduction in *snai2* and *tfap2e* expression, in 34.5% and 66% of the embryos, respectively (Figure 3B,C). Eftud2 knockdown reduced *snai2* in 26.7% of the embryos and did not affect *tfap2e* expression (Figure 2B,C), whereas Txnl4a knockdown caused a reduction in *tfap2e* expression in 32.4% of the embryos without affecting *snai2* (Figure 4B,C). Interestingly, *sox9*, which is acting upstream of *sox10* in the neural crest gene regulatory network, was largely unaffected in all three morphants (Figure 2B,C, Figure 3B,C and Figure 4B,C), suggesting that these factors may not regulate neural border specification but rather neural crest progenitor formation and/or maintenance, in a manner similar to that seen for the knockdown of another splicing factor, Sf3b4 [11]. To confirm the specificity of the morphant phenotypes we performed rescue experiments using constructs resistant to the MOs. In each case, the MO and rescuing DNA (100 pg) were co-injected at the two-cell stage and *sox10* expression was analyzed by ISH at stage 15. Injection of human EFTUD2 (HuEFTUD2) restored the proportion of embryos with normal *sox10* expression from 14% to 63% (Figure 2D,E), human SNRPB (HuSNRPB) from 16% to 41% (Figure 3D,E) and a modified version of *Xenopus Txnl4a* restored this proportion from 14% to 62% (Figure 4D,E). These results confirm the specificity of the phenotype for each knockdown, and indicate that Eftud2 and Snrpb have conserved function across species.

We expanded our analysis to include genes expressed in the developing cranial placodes, the precursors of sensory organs, as Sf3b4 knockdown also affected this cell population [26]. We found that the expression of the pan-placodal marker *six1* was reduced in embryos injected with Eftud2MO (37.7%), SnrpbMO (82%) and Txnl4MO (47.7%). The placode expression domain of *sox9* and *sox2* was also significantly reduced upon Eftud2 knockdown (56.8% and 50% of the embryos, respectively) or Snrpb knockdown (74% and 58% of the embryos, respectively), whereas Txnl4a morphant embryos showed reduced *sox9* and *sox2* expression in only 22.4% and 27.6% of injected embryos, respectively (Figure 2B,C, Figure 3B,C and Figure 4B,C). In all three morphant embryos the neural plate expression domain of *sox2* was marginally affected with approximately 10% of the embryos showing a mild expansion.

### 3.3. Increased TUNEL Positivity in Eftud2, Snrpb and Txnl4a Morphant Embryos

**Figure 5:**
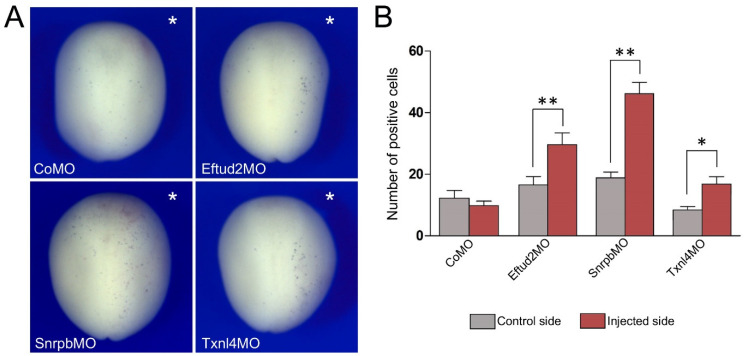
Eftud2, Snrpb and Txnl4a knockdown increase cell death in the ectoderm. (**A**) TUNEL staining of representative morphant embryos at stage 15. The injected side is indicated by an asterisk. Dorsal view, anterior to top. (**B**) Quantification of the number of TUNEL-positive cells in control vs. injected sides of embryos injected with Eftud2MO (n = 33), SnrpbMO (n = 39) and Txnl4MO (n = 41). Values are presented as mean ± s.e.m.; (*) *p*-value < 0.0005 and (**) *p*-value < 0.0001 (Student’s *t*-test).

As the expression of a number of genes critical for neural crest development was reduced in all three morphant embryos, we examined whether this reduction in gene expression correlated with a loss of neural crest progenitors through increased cell death. Embryos injected with CoMO (30 ng), Eftud2MO (30 ng), SnrpbMO (5 ng) or Txnl4MO (30 ng) were allowed to develop until stage 15 when apoptosis was assessed by TUNEL staining. For each embryo we quantified the number of TUNEL-positive cells in the dorsal ectoderm comparing control vs. injected sides for each MO. In all cases we observed a significant increase in the number of TUNEL-positive cells as a result of the three MOs’ injection, with the most significant increase observed for Snrpb knockdown (Figure 5A,B).

### 3.4. Eftud2, Snrpb and Txnl4a Knockdown Affect Craniofacial Development

**Figure 6:**
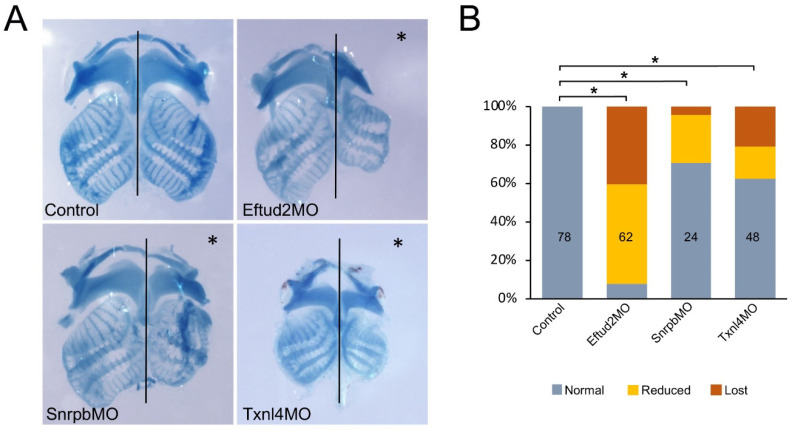
Eftud2, Snrpb and Txnl4a knockdown affect craniofacial cartilage formation. (**A**) Alcian blue staining of dissected craniofacial cartilages of control tadpoles and those injected with Eftud2MO (5 ng), SnrpbMO (1 ng) and Txnl4MO (30 ng), around stage 45. The injected side is indicted by an asterisk. The black lines indicate the midline. Note: Txnl4MO-injected tadpoles were collected at a slightly younger stage than their counterparts, which explains the overall size difference in craniofacial structures. (**B**) Quantification of the phenotypes. The numbers in each bar indicate the number of embryos analyzed. Control vs. MO-injected (*) *p*-value < 0.0001 (Chi-squared test).

We next analyzed the long-term effects of Eftud2, Snrpb and Txnl4a knockdown on the development of cranial neural-crest-derived cartilages at the tadpole stage using Alcian blue staining. Embryos injected with 30 ng Eftud2MO and 5 ng SnrpbMO did not survive to the tadpole stage; we therefore used lower concentrations for these MOs, 5 ng and 1 ng, respectively. The original concentration of Txnl4MO (30 ng) did not affect the long-term survival of these tadpoles. Eftud2 knockdown caused a reduction in or complete loss of cranial cartilages in 92% of injected tadpoles (Figure 6A,B), whereas Snrpb and Txnl4a knockdown produced cartilage defects at a lower frequency, in 29% and 38% of morphant tadpoles, respectively (Figure 6A,B).

## 4. Discussion

Spliceosomopathies define a category of diseases arising from mutations in genes encoding components of the spliceosome. They include three major conditions affecting primarily the retina (retinitis pigmentosa), bone marrow (myelodysplastic syndromes) and craniofacial skeleton (mandibulofacial dysostosis) [6,7,8,9]. It is unclear how protein variants that are functioning together as part of the same machinery can cause entirely distinct pathologies. It is also not well understood how defects in proteins presumably ubiquitously expressed can lead to cell-type-specific phenotypes. These questions underscore the important challenges that we face to understand these conditions, and the development of animal models represent an essential step in this direction.

Here, using *Xenopus laevis* we modeled three distinct craniofacial spliceosomopathies, MFDM (OMIM#610536), CCMS (OMIM#608572) and BMKS (OMIM#608572), that have been linked to haploinsufficient variants in EFTUD2 [27,28], SNRPB [29] and TXNL4A [30], respectively—three proteins associated with the U5 complex of the spliceosome. Our results indicate that in the absence of Eftud2, Snrpb or Txnl4a function there is a similar reduction in neural crest gene expression at the neural plate border, primarily affecting *sox10*, that correlates with an increased cell death in the ectoderm, and later translates into cranial cartilage defects. Although we did not evaluate p53 levels in morphant embryos, activation of this pathway in addition to increased apoptosis may also interfere with cell proliferation through activation of p21, a cell cycle inhibitor [31]. The knockdown phenotypes of Eftud2, Snrpb and Txnl4a are very similar to that of the animal model of NRS, another craniofacial splieceosomopathy, caused by haploinsufficiency of *SF3B4* [32,33,34,35], a protein associated with the U2 complex of the spliceosome. These animals also had reduced neural crest progenitors, increased apoptosis and hypoplastic cranial cartilages later in development [11]. Altogether, these findings suggest that MFD-derived spliceosomopathies share a common root cause, and that early depletion of cranial neural crest progenitors is the likely underlying mechanism causing MFD craniofacial abnormalities.

In zebrafish, three independent studies have reported the phenotypes of homozygous *eftud2* mutants. They are similarly characterized by small head and eyes and a curved body, and associated with dysplasia of neural-crest-derived head cartilages, with increased apoptosis, and altered proliferation in the developing head, pharyngeal arches and central nervous system, whereas heterozygous animals are normal [12,36,37]. In the mouse, Eftud2 heterozygous mutants were also normal at birth, whereas homozygous mutation resulted in embryonic lethality at the pre-implantation stage (Beauchamp et al., 2019). Conditional inactivation of Eftud2 in the neural crest lineage using the *Wnt1-Cre2* mouse line caused brain and midface abnormalities closely resembling MFDM. These malformations were associated with increased nuclear p53, enhanced expression of p53-activated genes and increased cell death, without affecting cell proliferation [13]. Human EFTUD2-knockdown in a cell line caused decreased proliferation and increased sensitivity to endoplasmic reticulum stress, promoting apoptosis. RNA-Seq analysis revealed widespread changes in gene expression with an enrichment for genes related to brain, cartilage and skeletal development [38]. Altogether these observations across species are consistent with the dysregulation of a neural crest and craniofacial developmental program as the primary cause for MFDM.

An in vitro model of BMKS was recently developed using patient-derived induced pluripotent stem cell (iPSC) lines [39]. Patient iPSCs exhibited slow proliferation rate and increased apoptosis as compared with control-derived iPSCs. Upon differentiation of the patient iPSCs into neural crest cells, RNA-Seq analysis revealed mis-splicing events preferentially affecting genes involved in neural crest specification as well as epithelial-to-mesenchymal transition, a fundamental process for the dissemination of neural crest cells in the embryo [39]. Models for other craniofacial spliceosomopathies such as CFM (OMIM#605591) and RCPS (OMIM#268305), which are due to mutations in *SF3B2* (a binding partner of SF3B4) and *EIF4A3* (a core component of the exon junction complex), respectively, also point to defects in neural crest development and function [40,41,42]. MO-mediated knockdown of *eif4a3* in zebrafish causes underdevelopment of craniofacial cartilages and clefting of the lower jaw, defects that are associated with increased cell death and reduced neural crest gene expression [40]. RCPS patient-derived iPSCs and mouse mutants both support a requirement of Eif4a3 in cranial neural crest cells and their derivatives, and in the regulation of osteochondrogenic differentiation during craniofacial development [42].

Although a consensus picture is starting to emerge on the underlying causes of craniofacial spliceosomopathies—the targeted depletion of neural crest progenitors and their derivatives through increased apoptosis—a number of important questions regarding the tissue specificity of these pathologies remain to be addressed. Interestingly, in frog, fish and mouse embryos, *Eftud2* transcripts are enriched in the developing head, brain and pharyngeal arches (Figure 1B; [17,43]). We also observed a similar expression pattern for *snrpb*, *txnl4a* and *sf3b4* in *Xenopus* (Figure 1B; [11]). In the mouse, Sf3b4 is expressed ubiquitously at all stages examined [44], whereas the expression of *Snrpb* and *Txnl4a* in other species has not been reported. This enriched expression of *Eftud2*, *Snrpb*, *Txnl4a* and *Sf3b4* in the developing head could account for the craniofacial-specific defects observed in the corresponding mutants. Recent evidence indicates that neural crest cells express higher levels of p53 than other embryonic cell types, and are therefore particularly sensitive to p53-mediated apoptosis, which could explain the cell-type-specificity of the phenotype [45]. Alternatively, the differential expression of unique interacting partners and/or target mRNAs in the neural crest lineage could explain the tissue-specificity of these pathologies. Future studies focusing on the characterization of these specific interactions may help resolve these outstanding questions.

## Data Availability

Data and reagents are available upon request.
